# Substitutional disorder in the substituted nixantphos ligand C_39_H_32_Br_0.27_Cl_0.73_NOP_2_

**DOI:** 10.1107/S1600536808007848

**Published:** 2008-04-02

**Authors:** Thashree Marimuthu, Muhammad D. Bala, Holger B. Friedrich

**Affiliations:** a School of Chemistry, University of KwaZulu-Natal, Westville Campus, Private Bag X54001, Durban 4000, South Africa

## Abstract

The structure of 10-(3-bromo/chloro­prop­yl)-4,6-bis­(diphenyl­phosphino)-10*H*-phenoxazine, C_39_H_32_Br_0.27_Cl_0.73_NOP_2_, shows chloro/bromo substitutional disorder in a 3:1 ratio. For application as a ligand in catalysis, the intra­molecular P⋯P distance of 4.263 (2) Å is relevant. The phenoxazine ring system is essentially planar.

## Related literature

For related literature see: Osiński *et al.* (2005 ▶); Ricken *et al.* (2006*a* ▶,*b* ▶); (Marimuthu *et al.*, 2008 ▶); Deprele & Montchamp (2004 ▶); Laungani *et al.* (2008 ▶); van Leeuwen *et al.* (2002 ▶); Norman *et al.* (2000 ▶); Ricken *et al.* (2006 ▶); Rotar *et al.* (2008 ▶); Sandee *et al.* (1999 ▶, 2001 ▶); Web *et al.* (2005 ▶).
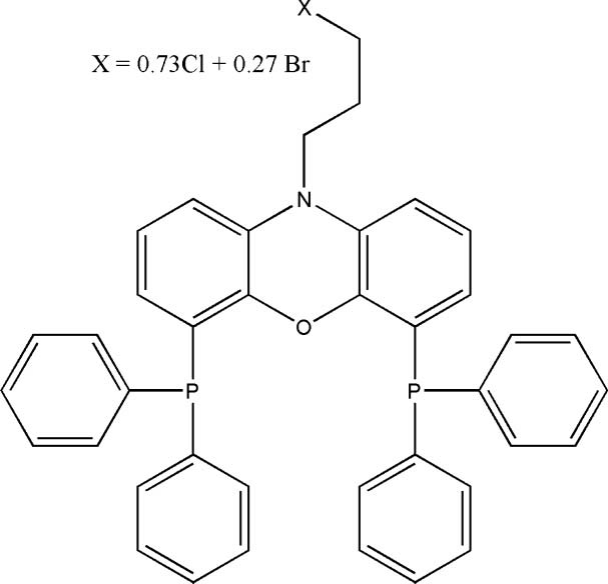


## Experimental

### 

#### Crystal data

C_39_H_32_Br_0.27_Cl_0.73_NOP_2_
*M* *_r_* = 639.83
Triclinic, 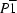

*a* = 10.0539 (3) Å
*b* = 11.4469 (3) Å
*c* = 14.5299 (3) Å
α = 69.544 (1)°
β = 83.283 (2)°
γ = 81.453 (1)°
*V* = 1545.52 (7) Å^3^
*Z* = 2
Mo *K*α radiation
μ = 0.58 mm^−1^
*T* = 173 (2) K
0.42 × 0.19 × 0.17 mm

#### Data collection

Bruker APEXII CCD area-detector diffractometer
Absorption correction: integration (*XPREP*; Bruker, 2005 ▶) *T*_min_ = 0.792, *T*_max_ = 0.908
24195 measured reflections
7458 independent reflections
5180 reflections with *I* > 2σ(*I*)
*R*_int_ = 0.051

#### Refinement

*R*[*F* ^2^ > 2σ(*F* ^2^)] = 0.041
*wR*(*F* ^2^) = 0.097
*S* = 0.94
7458 reflections
401 parameters
2 restraints
H-atom parameters constrained
Δρ_max_ = 0.36 e Å^−3^
Δρ_min_ = −0.28 e Å^−3^

### 

Data collection: *APEX2* (Bruker, 2005 ▶); cell refinement: *SAINT* (Bruker, 2005 ▶); data reduction: *SAINT*; program(s) used to solve structure: *SHELXTL* (Sheldrick, 2008 ▶); program(s) used to refine structure: *SHELXTL*; molecular graphics: *PLATON* (Spek, 2003 ▶) and *ORTEP-3 for Windows* (Farrugia, 1997 ▶); software used to prepare material for publication: *SHELXTL*.

## Supplementary Material

Crystal structure: contains datablocks I. DOI: 10.1107/S1600536808007848/pv2073sup1.cif

Structure factors: contains datablocks I. DOI: 10.1107/S1600536808007848/pv2073Isup2.hkl

Additional supplementary materials:  crystallographic information; 3D view; checkCIF report

## Figures and Tables

**Fig. 1.**
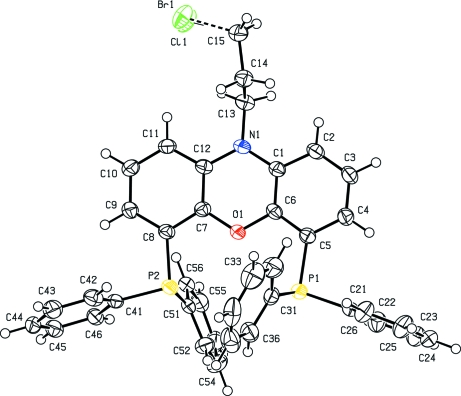
Molecular structure of (I) with thermal ellipsoids shown at 50% probability levels.

## supplementary crystallographic information

### Comment

As part of our ongoing investigation of scorpionate-type ligands (Marimuthu *et al.*, 2008) the title ligand, a diphenylphosphine xanthene based compound, (I) has been prepared in our laboratory. The synthesis strategy involved the addition of an alkyl chain to the amine to form a tridentate ligand. Unlike other scorpionate ligands, (I) has a mixed donor arrangement consisting of N, O & P and it has been used as a precursor in the synthesis of modified nixantphos ligands for continuous flow homogenous hydroformylation of alkenes using supercritical fluids (Web *et al.*, 2005). The functionalization of the nitrogen has been reported in the literature, where the subsequent compounds were successfully immobilized on silica (Sandee *et al.*, 1999, 2001; van Leeuwen *et al.*, 2002), polystyrene (Deprele & Montchamp, 2004) and dendritic supports (Ricken *et al.*, 2006).

The structure of (I) contains chloro- and bromo- substituted alkyl chains in 3:1 ratio (Fig. 1) with an essentially planar phenoxazine ring [with maximum deviation of 0.113 (3) Å for O1] joining the two diphenylphosphino groups. The intramolecular P—P distance of 4.263 (2) Å in (I) compares well to 4.255 (2) Å reported for the precursor nixantphos (Marimuthu *et al.*, 2008). The bond lengths for C—O range from 1.380 (2) to 1.384 (2) and for C—N from 1.398 (2) to 1.402 (2) Å. The bond angles involving the P atoms range from 100.52 (7) to 102.11 (8)°.

The substitutional disorder observed in the halide site of (I) was modelled as 3:1 Cl to Br. The source of the disorder is the sodium hydride induced alkylation using 1-bromo-3-chloropropane adapted for this work. The ligand 1-bromo-3-chloropropane is symmetrical with a chloride and a bromide functionality on either side of a propyl chain, hence a competitive substitution reaction between the two leaving groups. The model of the disordered halide site is in agreement with Br as the more basic and better leaving group than Cl. Other cases of halide substitutional disorder have been reported in the literature (Norman *et al.*, 2000; Laungani *et al.*, 2008; Rotar *et al.*, 2008) and it is interesting to note that all contain only Cl/Br disorder in the 3:1 ratio.

### Experimental

Synthesis adapted from literature (Web *et al.*, 2005). Yield: 74% of colourless crystals of (I) grown from a solution of dichloromethane/ethanol (1:1) at room temperature, m.p. 493 K (dec.).

### Refinement

All hydrogen atoms were located from a difference map then positioned geometrically and allowed to ride on their respective parent atoms (C—H = 0.95 - 0.99 Å) with *U*_iso_(H) = 1.2 *U*_eq_(C) for aryl H or 1.5 *U*_eq_(C) for CH_2_. The structure contains substitutional disorder in which Cl1 and Br1 occupy the same position. These were refined with C—Cl and C—Br distances restrained to 1.78 and 1.93 Å and final occupancies for Cl1 and Br1 were 0.734 (2) and 0.266 (2), respectively.

### Crystal data

**Table:**

C_39_H_32_Br_0.27_Cl_0.73_NOP_2_	*Z* = 2
*M**_r_* = 639.83	*F*_000_ = 665.6
Triclinic, *P*1	*D*_x_ = 1.375 Mg m^−^^3^
Hall symbol: -P 1	Melting point: 493(2) K
*a* = 10.0539 (3) Å	Mo *K*α radiation λ = 0.71073 Å
*b* = 11.4469 (3) Å	Cell parameters from 6264 reflections
*c* = 14.5299 (3) Å	θ = 2.5–28.2º
α = 69.544 (1)º	µ = 0.58 mm^−^^1^
β = 83.283 (2)º	*T* = 173 (2) K
γ = 81.453 (1)º	Prism, colourless
*V* = 1545.52 (7) Å^3^	0.42 × 0.19 × 0.17 mm

### Data collection

**Table:**

Bruker APEXII CCD area-detector diffractometer	7458 independent reflections
Radiation source: fine-focus sealed tube	5180 reflections with *I* > 2σ(*I*)
Monochromator: graphite	*R*_int_ = 0.051
*T* = 173(2) K	θ_max_ = 28.0º
φ and ω scans	θ_min_ = 1.5º
Absorption correction: integration(XPREP; Bruker, 2005)	*h* = −13→12
*T*_min_ = 0.792, *T*_max_ = 0.908	*k* = −15→15
24195 measured reflections	*l* = −17→19

### Refinement

**Table:**

Refinement on *F*^2^	Secondary atom site location: difference Fourier map
Least-squares matrix: full	Hydrogen site location: inferred from neighbouring sites
*R*[*F*^2^ > 2σ(*F*^2^)] = 0.041	H-atom parameters constrained
*wR*(*F*^2^) = 0.097	*w* = 1/[σ^2^(*F*_o_^2^) + (0.0499*P*)^2^] where *P* = (*F*_o_^2^ + 2*F*_c_^2^)/3
*S* = 0.94	(Δ/σ)_max_ = 0.006
7458 reflections	Δρ_max_ = 0.36 e Å^−^^3^
401 parameters	Δρ_min_ = −0.28 e Å^−^^3^
2 restraints	Extinction correction: none
Primary atom site location: structure-invariant direct methods	

### Special details

**Table:**

Experimental. Spectroscopic analysis: ^1^H NMR (400 MHz,CDCl_3_, δ, p.p.m.) = 2.13(m, 2H; CH), 2.16(m, 2H; CH), 3.52(m, 2H; CH), 3.69(m, 2H; CH), 6.0(d, 2H; J(H,H) = 7.5 Hz,), 6.34(bd, 2H; J(H,H) = 7.5 Hz,), 6.65(t, 2H J(H,H) = 7.8 Hz), 7.18 – 7.21(bs, 20H); ^13^C NMR (400 MHz, CDCl_3_, δ, p.p.m.); = 27.5(ClCH_2_), 30.8(BrCH_2_), 41.8(NCH_2_), 42.6(NCH_2_), 42.6(NCH_2_), 111.6(CH), 123.7(CH), 125.5 (C), 128.1(CH), 132.8(CN), 133.8(C),136.8(C),147.0(CO) ^31^P NMR (600 MHz,CDCl_3_, δ, p.p.m.) = -19.0; FTIR: cm^-1^ = 1552(*s*), 1462(*s*), 1433(*s*), 1418(*s*), 1380(*s*), 1274(s CN), 1257(*m*),1206(*m*), 1092(*m*), 765(*m*), 747(*m*), 722(*m*), 697(*s*).516(*s*), 496(*s*), 433(*s*), 400(*s*); Calculated for C_39_H_32_Br_0.27_Cl_0.73_NOP_2_: C=72.03; H= 4.96; N= 2.15; Found C=71.55; H= 4.95; N= 2.26.
Geometry. All e.s.d.'s (except the e.s.d. in the dihedral angle between two l.s. planes) are estimated using the full covariance matrix. The cell e.s.d.'s are taken into account individually in the estimation of e.s.d.'s in distances, angles and torsion angles; correlations between e.s.d.'s in cell parameters are only used when they are defined by crystal symmetry. An approximate (isotropic) treatment of cell e.s.d.'s is used for estimating e.s.d.'s involving l.s. planes.
Refinement. Refinement of *F*^2^ against ALL reflections. The weighted *R*-factor *wR* and goodness of fit *S* are based on *F*^2^, conventional *R*-factors *R* are based on *F*, with *F* set to zero for negative *F*^2^. The threshold expression of *F*^2^ > σ(*F*^2^) is used only for calculating *R*-factors(gt) *etc*. and is not relevant to the choice of reflections for refinement. *R*-factors based on *F*^2^ are statistically about twice as large as those based on *F*, and *R*- factors based on ALL data will be even larger.

### Fractional atomic coordinates and isotropic or equivalent isotropic displacement parameters (Å^2^)

**Table:**

	*x*	*y*	*z*	*U*_iso_*/*U*_eq_	Occ. (<1)
C1	0.12106 (16)	0.58921 (15)	0.41860 (12)	0.0285 (4)	
C2	0.06839 (17)	0.70497 (16)	0.42572 (13)	0.0337 (4)	
H2	0.0852	0.7255	0.4809	0.040*	
C3	−0.00868 (18)	0.79126 (17)	0.35303 (13)	0.0360 (4)	
H3	−0.0466	0.8690	0.3601	0.043*	
C4	−0.03072 (17)	0.76542 (16)	0.27087 (13)	0.0326 (4)	
H4	−0.0819	0.8261	0.2210	0.039*	
C5	0.02184 (16)	0.65037 (15)	0.26046 (12)	0.0275 (4)	
C6	0.09436 (16)	0.56450 (15)	0.33551 (12)	0.0285 (4)	
C7	0.22129 (16)	0.36362 (15)	0.39245 (11)	0.0278 (4)	
C8	0.26881 (17)	0.25469 (15)	0.37271 (12)	0.0283 (4)	
C9	0.35333 (18)	0.16430 (16)	0.43904 (12)	0.0349 (4)	
H9	0.3863	0.0875	0.4285	0.042*	
C10	0.38889 (19)	0.18641 (17)	0.51980 (13)	0.0376 (4)	
H10	0.4471	0.1249	0.5642	0.045*	
C11	0.34067 (17)	0.29726 (16)	0.53695 (12)	0.0322 (4)	
H11	0.3677	0.3119	0.5921	0.039*	
C12	0.25368 (16)	0.38670 (15)	0.47466 (12)	0.0284 (4)	
C13	0.22292 (18)	0.52225 (17)	0.57888 (12)	0.0348 (4)	
H13A	0.2434	0.4414	0.6323	0.042*	
H13B	0.1411	0.5677	0.6012	0.042*	
C14	0.33988 (17)	0.59931 (17)	0.56022 (12)	0.0348 (4)	
H14A	0.4163	0.5629	0.5255	0.042*	
H14B	0.3124	0.6860	0.5168	0.042*	
C15	0.38590 (18)	0.60363 (13)	0.65430 (13)	0.0417 (5)	
H15A	0.3061	0.6182	0.6975	0.050*	
H15B	0.4399	0.6746	0.6385	0.050*	
C21	−0.10597 (16)	0.74294 (16)	0.08074 (12)	0.0288 (4)	
C22	−0.24546 (18)	0.75239 (18)	0.09281 (14)	0.0403 (4)	
H22	−0.2878	0.6862	0.1412	0.048*	
C23	−0.32390 (19)	0.8556 (2)	0.03611 (15)	0.0451 (5)	
H23	−0.4193	0.8604	0.0467	0.054*	
C24	−0.26619 (19)	0.95128 (18)	−0.03519 (13)	0.0409 (5)	
H24	−0.3207	1.0222	−0.0746	0.049*	
C25	−0.1281 (2)	0.9435 (2)	−0.04912 (14)	0.0481 (5)	
H25	−0.0867	1.0091	−0.0988	0.058*	
C26	−0.04918 (19)	0.84080 (18)	0.00873 (13)	0.0420 (5)	
H26	0.0462	0.8374	−0.0012	0.050*	
C31	0.15173 (17)	0.60609 (16)	0.08889 (12)	0.0327 (4)	
C32	0.24948 (19)	0.6776 (2)	0.09138 (15)	0.0489 (5)	
H32	0.2373	0.7233	0.1357	0.059*	
C33	0.3658 (2)	0.6825 (2)	0.02890 (18)	0.0614 (6)	
H33	0.4328	0.7318	0.0307	0.074*	
C34	0.3846 (2)	0.6171 (2)	−0.03508 (16)	0.0588 (6)	
H34	0.4641	0.6218	−0.0780	0.071*	
C35	0.2896 (2)	0.5453 (2)	−0.03736 (16)	0.0567 (6)	
H35	0.3026	0.4997	−0.0818	0.068*	
C36	0.1751 (2)	0.53893 (18)	0.02441 (14)	0.0433 (5)	
H36	0.1102	0.4873	0.0231	0.052*	
C41	0.32148 (18)	0.09367 (15)	0.26043 (11)	0.0299 (4)	
C42	0.45411 (19)	0.09954 (17)	0.22056 (13)	0.0374 (4)	
H42	0.4897	0.1779	0.1972	0.045*	
C43	0.53459 (19)	−0.00586 (19)	0.21437 (14)	0.0418 (5)	
H43	0.6251	0.0000	0.1874	0.050*	
C44	0.48400 (19)	−0.12036 (17)	0.24733 (13)	0.0380 (4)	
H44	0.5392	−0.1932	0.2425	0.046*	
C45	0.35297 (19)	−0.12821 (17)	0.28719 (12)	0.0370 (4)	
H45	0.3178	−0.2068	0.3102	0.044*	
C46	0.27231 (18)	−0.02186 (15)	0.29384 (12)	0.0318 (4)	
H46	0.1822	−0.0282	0.3216	0.038*	
C51	0.05273 (18)	0.19766 (15)	0.29170 (13)	0.0322 (4)	
C52	−0.0264 (2)	0.21254 (18)	0.21515 (14)	0.0445 (5)	
H52	0.0075	0.2503	0.1491	0.053*	
C53	−0.1533 (2)	0.1735 (2)	0.23338 (17)	0.0534 (6)	
H53	−0.2046	0.1821	0.1800	0.064*	
C54	−0.2052 (2)	0.12224 (19)	0.32848 (17)	0.0494 (5)	
H54	−0.2922	0.0946	0.3411	0.059*	
C55	−0.13087 (19)	0.11098 (18)	0.40537 (15)	0.0423 (5)	
H55	−0.1678	0.0782	0.4713	0.051*	
C56	−0.00240 (18)	0.14728 (16)	0.38703 (13)	0.0347 (4)	
H56	0.0488	0.1375	0.4407	0.042*	
N1	0.19570 (15)	0.49747 (13)	0.49120 (10)	0.0332 (3)	
O1	0.13571 (13)	0.44822 (11)	0.32597 (9)	0.0371 (3)	
P1	−0.01140 (4)	0.60026 (4)	0.15924 (3)	0.02944 (12)	
P2	0.22335 (5)	0.24299 (4)	0.25806 (3)	0.03152 (12)	
Cl1	0.4842 (12)	0.4617 (8)	0.7179 (10)	0.0488 (6)	0.7338 (16)
Br1	0.4972 (14)	0.4545 (9)	0.7259 (12)	0.0488 (6)	0.2662 (16)

### Atomic displacement parameters (Å^2^)

**Table:**

	*U*^11^	*U*^22^	*U*^33^	*U*^12^	*U*^13^	*U*^23^
C1	0.0291 (9)	0.0290 (9)	0.0308 (9)	−0.0055 (7)	−0.0015 (7)	−0.0135 (7)
C2	0.0349 (10)	0.0347 (10)	0.0387 (10)	−0.0040 (8)	−0.0022 (8)	−0.0215 (8)
C3	0.0360 (10)	0.0317 (10)	0.0461 (11)	−0.0001 (8)	−0.0033 (8)	−0.0218 (8)
C4	0.0333 (9)	0.0284 (9)	0.0370 (9)	−0.0020 (8)	−0.0033 (7)	−0.0125 (8)
C5	0.0294 (9)	0.0250 (9)	0.0293 (8)	−0.0063 (7)	−0.0010 (7)	−0.0096 (7)
C6	0.0309 (9)	0.0251 (9)	0.0332 (9)	−0.0056 (7)	−0.0009 (7)	−0.0139 (7)
C7	0.0318 (9)	0.0251 (9)	0.0271 (8)	−0.0061 (7)	−0.0048 (7)	−0.0076 (7)
C8	0.0349 (9)	0.0243 (9)	0.0277 (8)	−0.0056 (7)	−0.0045 (7)	−0.0097 (7)
C9	0.0475 (11)	0.0240 (9)	0.0343 (9)	−0.0010 (8)	−0.0101 (8)	−0.0102 (7)
C10	0.0457 (11)	0.0325 (10)	0.0342 (10)	−0.0007 (8)	−0.0143 (8)	−0.0089 (8)
C11	0.0397 (10)	0.0321 (10)	0.0277 (9)	−0.0049 (8)	−0.0082 (7)	−0.0116 (7)
C12	0.0328 (9)	0.0281 (9)	0.0274 (8)	−0.0082 (7)	−0.0016 (7)	−0.0115 (7)
C13	0.0417 (10)	0.0361 (10)	0.0306 (9)	−0.0086 (8)	−0.0008 (8)	−0.0151 (8)
C14	0.0375 (10)	0.0350 (10)	0.0325 (9)	−0.0063 (8)	−0.0014 (8)	−0.0117 (8)
C15	0.0490 (12)	0.0382 (11)	0.0431 (11)	−0.0037 (9)	−0.0135 (9)	−0.0175 (9)
C21	0.0313 (9)	0.0306 (9)	0.0284 (9)	−0.0019 (7)	−0.0044 (7)	−0.0146 (7)
C22	0.0340 (10)	0.0389 (11)	0.0462 (11)	−0.0041 (9)	−0.0019 (8)	−0.0125 (9)
C23	0.0313 (10)	0.0525 (13)	0.0550 (12)	0.0018 (9)	−0.0081 (9)	−0.0236 (10)
C24	0.0470 (12)	0.0409 (11)	0.0354 (10)	0.0077 (9)	−0.0160 (9)	−0.0148 (9)
C25	0.0473 (12)	0.0459 (12)	0.0393 (11)	−0.0031 (10)	−0.0063 (9)	0.0004 (9)
C26	0.0334 (10)	0.0459 (12)	0.0394 (10)	−0.0040 (9)	−0.0043 (8)	−0.0050 (9)
C31	0.0317 (9)	0.0314 (10)	0.0314 (9)	0.0029 (8)	−0.0066 (7)	−0.0073 (8)
C32	0.0375 (11)	0.0573 (14)	0.0555 (13)	−0.0081 (10)	−0.0035 (9)	−0.0223 (11)
C33	0.0393 (12)	0.0654 (16)	0.0711 (16)	−0.0138 (11)	−0.0013 (11)	−0.0104 (13)
C34	0.0501 (14)	0.0506 (14)	0.0515 (13)	0.0140 (11)	0.0096 (10)	0.0004 (11)
C35	0.0631 (15)	0.0510 (14)	0.0477 (13)	0.0068 (12)	0.0093 (11)	−0.0159 (11)
C36	0.0486 (12)	0.0397 (11)	0.0410 (11)	0.0024 (9)	−0.0024 (9)	−0.0164 (9)
C41	0.0419 (10)	0.0256 (9)	0.0243 (8)	−0.0011 (8)	−0.0082 (7)	−0.0104 (7)
C42	0.0447 (11)	0.0349 (10)	0.0362 (10)	−0.0095 (9)	−0.0031 (8)	−0.0145 (8)
C43	0.0369 (10)	0.0507 (13)	0.0406 (11)	−0.0016 (9)	−0.0034 (8)	−0.0201 (9)
C44	0.0480 (11)	0.0346 (10)	0.0310 (9)	0.0104 (9)	−0.0113 (8)	−0.0140 (8)
C45	0.0548 (12)	0.0267 (9)	0.0287 (9)	−0.0029 (9)	−0.0051 (8)	−0.0082 (7)
C46	0.0409 (10)	0.0268 (9)	0.0278 (9)	−0.0024 (8)	−0.0019 (7)	−0.0100 (7)
C51	0.0421 (10)	0.0230 (9)	0.0366 (10)	0.0056 (8)	−0.0128 (8)	−0.0169 (7)
C52	0.0585 (13)	0.0376 (11)	0.0401 (11)	−0.0005 (10)	−0.0214 (9)	−0.0132 (9)
C53	0.0552 (13)	0.0475 (13)	0.0629 (14)	0.0005 (11)	−0.0333 (11)	−0.0189 (11)
C54	0.0403 (11)	0.0394 (12)	0.0740 (15)	0.0032 (9)	−0.0180 (11)	−0.0245 (11)
C55	0.0416 (11)	0.0380 (11)	0.0513 (12)	0.0013 (9)	−0.0038 (9)	−0.0222 (9)
C56	0.0386 (10)	0.0333 (10)	0.0382 (10)	0.0034 (8)	−0.0103 (8)	−0.0201 (8)
N1	0.0436 (9)	0.0305 (8)	0.0312 (8)	−0.0019 (7)	−0.0088 (7)	−0.0167 (6)
O1	0.0524 (8)	0.0265 (6)	0.0376 (7)	0.0075 (6)	−0.0200 (6)	−0.0169 (5)
P1	0.0326 (2)	0.0276 (2)	0.0302 (2)	−0.00380 (19)	−0.00480 (18)	−0.01137 (19)
P2	0.0463 (3)	0.0230 (2)	0.0269 (2)	−0.0012 (2)	−0.0079 (2)	−0.00993 (18)
Cl1	0.0433 (17)	0.0434 (7)	0.0459 (17)	−0.0032 (4)	−0.0128 (10)	0.0046 (7)
Br1	0.0433 (17)	0.0434 (7)	0.0459 (17)	−0.0032 (4)	−0.0128 (10)	0.0046 (7)

### Geometric parameters (Å, °)

**Table:**

C1—C2	1.386 (2)	C24—C25	1.373 (3)
C1—C6	1.395 (2)	C24—H24	0.9500
C1—N1	1.398 (2)	C25—C26	1.380 (3)
C2—C3	1.388 (2)	C25—H25	0.9500
C2—H2	0.9500	C26—H26	0.9500
C3—C4	1.375 (2)	C31—C32	1.380 (3)
C3—H3	0.9500	C31—C36	1.386 (3)
C4—C5	1.396 (2)	C31—P1	1.8253 (18)
C4—H4	0.9500	C32—C33	1.389 (3)
C5—C6	1.382 (2)	C32—H32	0.9500
C5—P1	1.8345 (16)	C33—C34	1.365 (3)
C6—O1	1.3837 (19)	C33—H33	0.9500
C7—O1	1.3799 (19)	C34—C35	1.360 (3)
C7—C8	1.381 (2)	C34—H34	0.9500
C7—C12	1.393 (2)	C35—C36	1.368 (3)
C8—C9	1.398 (2)	C35—H35	0.9500
C8—P2	1.8313 (16)	C36—H36	0.9500
C9—C10	1.379 (2)	C41—C46	1.385 (2)
C9—H9	0.9500	C41—C42	1.392 (2)
C10—C11	1.385 (2)	C41—P2	1.8335 (17)
C10—H10	0.9500	C42—C43	1.374 (3)
C11—C12	1.380 (2)	C42—H42	0.9500
C11—H11	0.9500	C43—C44	1.381 (3)
C12—N1	1.402 (2)	C43—H43	0.9500
C13—N1	1.461 (2)	C44—C45	1.378 (3)
C13—C14	1.517 (2)	C44—H44	0.9500
C13—H13A	0.9900	C45—C46	1.385 (2)
C13—H13B	0.9900	C45—H45	0.9500
C14—C15	1.512 (2)	C46—H46	0.9500
C14—H14A	0.9900	C51—C56	1.384 (2)
C14—H14B	0.9900	C51—C52	1.390 (2)
C15—Cl1	1.7770 (18)	C51—P2	1.8281 (18)
C15—Br1	1.9266 (19)	C52—C53	1.380 (3)
C15—H15A	0.9900	C52—H52	0.9500
C15—H15B	0.9900	C53—C54	1.372 (3)
C21—C26	1.381 (2)	C53—H53	0.9500
C21—C22	1.386 (2)	C54—C55	1.374 (3)
C21—P1	1.8338 (17)	C54—H54	0.9500
C22—C23	1.375 (3)	C55—C56	1.383 (2)
C22—H22	0.9500	C55—H55	0.9500
C23—C24	1.365 (3)	C56—H56	0.9500
C23—H23	0.9500		
			
C2—C1—C6	117.26 (15)	C24—C25—H25	119.8
C2—C1—N1	122.98 (15)	C26—C25—H25	119.8
C6—C1—N1	119.74 (15)	C25—C26—C21	121.39 (18)
C1—C2—C3	120.70 (16)	C25—C26—H26	119.3
C1—C2—H2	119.7	C21—C26—H26	119.3
C3—C2—H2	119.7	C32—C31—C36	118.05 (18)
C4—C3—C2	120.72 (17)	C32—C31—P1	124.77 (15)
C4—C3—H3	119.6	C36—C31—P1	117.08 (14)
C2—C3—H3	119.6	C31—C32—C33	119.9 (2)
C3—C4—C5	120.29 (17)	C31—C32—H32	120.1
C3—C4—H4	119.9	C33—C32—H32	120.1
C5—C4—H4	119.9	C34—C33—C32	120.7 (2)
C6—C5—C4	117.78 (15)	C34—C33—H33	119.7
C6—C5—P1	117.69 (12)	C32—C33—H33	119.7
C4—C5—P1	124.31 (13)	C35—C34—C33	119.9 (2)
C5—C6—O1	115.95 (14)	C35—C34—H34	120.0
C5—C6—C1	123.20 (15)	C33—C34—H34	120.0
O1—C6—C1	120.80 (15)	C34—C35—C36	119.9 (2)
O1—C7—C8	115.32 (14)	C34—C35—H35	120.0
O1—C7—C12	121.59 (15)	C36—C35—H35	120.0
C8—C7—C12	123.07 (15)	C35—C36—C31	121.5 (2)
C7—C8—C9	117.72 (15)	C35—C36—H36	119.2
C7—C8—P2	117.39 (12)	C31—C36—H36	119.2
C9—C8—P2	124.82 (13)	C46—C41—C42	118.10 (16)
C10—C9—C8	120.13 (16)	C46—C41—P2	125.35 (14)
C10—C9—H9	119.9	C42—C41—P2	116.47 (13)
C8—C9—H9	119.9	C43—C42—C41	121.27 (17)
C9—C10—C11	120.73 (17)	C43—C42—H42	119.4
C9—C10—H10	119.6	C41—C42—H42	119.4
C11—C10—H10	119.6	C42—C43—C44	120.04 (18)
C12—C11—C10	120.61 (15)	C42—C43—H43	120.0
C12—C11—H11	119.7	C44—C43—H43	120.0
C10—C11—H11	119.7	C45—C44—C43	119.59 (17)
C11—C12—C7	117.69 (16)	C45—C44—H44	120.2
C11—C12—N1	123.19 (14)	C43—C44—H44	120.2
C7—C12—N1	119.11 (15)	C44—C45—C46	120.22 (17)
N1—C13—C14	112.39 (14)	C44—C45—H45	119.9
N1—C13—H13A	109.1	C46—C45—H45	119.9
C14—C13—H13A	109.1	C41—C46—C45	120.78 (17)
N1—C13—H13B	109.1	C41—C46—H46	119.6
C14—C13—H13B	109.1	C45—C46—H46	119.6
H13A—C13—H13B	107.9	C56—C51—C52	117.67 (17)
C15—C14—C13	112.46 (15)	C56—C51—P2	125.18 (13)
C15—C14—H14A	109.1	C52—C51—P2	117.13 (14)
C13—C14—H14A	109.1	C53—C52—C51	121.25 (19)
C15—C14—H14B	109.1	C53—C52—H52	119.4
C13—C14—H14B	109.1	C51—C52—H52	119.4
H14A—C14—H14B	107.8	C54—C53—C52	120.04 (18)
C14—C15—Cl1	111.5 (5)	C54—C53—H53	120.0
C14—C15—Br1	114.0 (6)	C52—C53—H53	120.0
C14—C15—H15A	109.3	C53—C54—C55	119.73 (19)
Cl1—C15—H15A	109.3	C53—C54—H54	120.1
Br1—C15—H15A	109.2	C55—C54—H54	120.1
C14—C15—H15B	109.3	C54—C55—C56	120.16 (19)
Cl1—C15—H15B	109.3	C54—C55—H55	119.9
Br1—C15—H15B	106.8	C56—C55—H55	119.9
H15A—C15—H15B	108.0	C55—C56—C51	121.07 (17)
C26—C21—C22	117.21 (17)	C55—C56—H56	119.5
C26—C21—P1	125.12 (13)	C51—C56—H56	119.5
C22—C21—P1	117.65 (13)	C1—N1—C12	119.14 (13)
C23—C22—C21	121.32 (17)	C1—N1—C13	120.04 (14)
C23—C22—H22	119.3	C12—N1—C13	120.59 (14)
C21—C22—H22	119.3	C7—O1—C6	118.58 (12)
C24—C23—C22	120.74 (18)	C31—P1—C21	100.52 (7)
C24—C23—H23	119.6	C31—P1—C5	102.11 (8)
C22—C23—H23	119.6	C21—P1—C5	101.24 (7)
C23—C24—C25	118.97 (18)	C51—P2—C8	102.00 (8)
C23—C24—H24	120.5	C51—P2—C41	101.66 (8)
C25—C24—H24	120.5	C8—P2—C41	100.58 (7)
C24—C25—C26	120.35 (18)		
			
C6—C1—C2—C3	0.6 (2)	C43—C44—C45—C46	0.2 (3)
N1—C1—C2—C3	−177.93 (15)	C42—C41—C46—C45	−0.3 (2)
C1—C2—C3—C4	−2.2 (3)	P2—C41—C46—C45	176.23 (13)
C2—C3—C4—C5	1.4 (3)	C44—C45—C46—C41	0.2 (3)
C3—C4—C5—C6	0.7 (2)	C56—C51—C52—C53	−2.7 (3)
C3—C4—C5—P1	175.10 (13)	P2—C51—C52—C53	175.83 (15)
C4—C5—C6—O1	175.16 (14)	C51—C52—C53—C54	1.9 (3)
P1—C5—C6—O1	0.4 (2)	C52—C53—C54—C55	0.6 (3)
C4—C5—C6—C1	−2.3 (2)	C53—C54—C55—C56	−2.2 (3)
P1—C5—C6—C1	−177.06 (13)	C54—C55—C56—C51	1.3 (3)
C2—C1—C6—C5	1.6 (2)	C52—C51—C56—C55	1.1 (3)
N1—C1—C6—C5	−179.76 (15)	P2—C51—C56—C55	−177.33 (14)
C2—C1—C6—O1	−175.72 (15)	C2—C1—N1—C12	−175.29 (15)
N1—C1—C6—O1	2.9 (2)	C6—C1—N1—C12	6.2 (2)
O1—C7—C8—C9	−178.47 (14)	C2—C1—N1—C13	−0.9 (2)
C12—C7—C8—C9	0.0 (2)	C6—C1—N1—C13	−179.40 (14)
O1—C7—C8—P2	4.6 (2)	C11—C12—N1—C1	172.78 (15)
C12—C7—C8—P2	−176.93 (12)	C7—C12—N1—C1	−8.2 (2)
C7—C8—C9—C10	−1.3 (2)	C11—C12—N1—C13	−1.6 (2)
P2—C8—C9—C10	175.33 (14)	C7—C12—N1—C13	177.40 (14)
C8—C9—C10—C11	0.7 (3)	C14—C13—N1—C1	−80.73 (19)
C9—C10—C11—C12	1.4 (3)	C14—C13—N1—C12	93.60 (19)
C10—C11—C12—C7	−2.7 (2)	C8—C7—O1—C6	−173.86 (14)
C10—C11—C12—N1	176.35 (16)	C12—C7—O1—C6	7.7 (2)
O1—C7—C12—C11	−179.65 (14)	C5—C6—O1—C7	172.70 (14)
C8—C7—C12—C11	2.0 (2)	C1—C6—O1—C7	−9.8 (2)
O1—C7—C12—N1	1.3 (2)	C32—C31—P1—C21	81.38 (17)
C8—C7—C12—N1	−177.05 (15)	C36—C31—P1—C21	−94.87 (15)
N1—C13—C14—C15	−168.77 (14)	C32—C31—P1—C5	−22.66 (18)
C13—C14—C15—Cl1	77.1 (5)	C36—C31—P1—C5	161.09 (14)
C13—C14—C15—Br1	78.6 (6)	C26—C21—P1—C31	−17.74 (17)
C26—C21—C22—C23	−0.7 (3)	C22—C21—P1—C31	160.68 (14)
P1—C21—C22—C23	−179.23 (15)	C26—C21—P1—C5	87.00 (16)
C21—C22—C23—C24	1.1 (3)	C22—C21—P1—C5	−94.58 (14)
C22—C23—C24—C25	−0.5 (3)	C6—C5—P1—C31	−76.01 (14)
C23—C24—C25—C26	−0.5 (3)	C4—C5—P1—C31	109.61 (15)
C24—C25—C26—C21	0.9 (3)	C6—C5—P1—C21	−179.49 (13)
C22—C21—C26—C25	−0.3 (3)	C4—C5—P1—C21	6.13 (16)
P1—C21—C26—C25	178.11 (15)	C56—C51—P2—C8	−18.19 (16)
C36—C31—C32—C33	1.3 (3)	C52—C51—P2—C8	163.35 (14)
P1—C31—C32—C33	−174.94 (16)	C56—C51—P2—C41	85.44 (16)
C31—C32—C33—C34	−0.1 (3)	C52—C51—P2—C41	−93.02 (14)
C32—C33—C34—C35	−0.6 (3)	C7—C8—P2—C51	−80.83 (14)
C33—C34—C35—C36	0.1 (3)	C9—C8—P2—C51	102.50 (15)
C34—C35—C36—C31	1.2 (3)	C7—C8—P2—C41	174.69 (13)
C32—C31—C36—C35	−1.8 (3)	C9—C8—P2—C41	−1.97 (16)
P1—C31—C36—C35	174.67 (16)	C46—C41—P2—C51	−7.91 (16)
C46—C41—C42—C43	−0.1 (3)	C42—C41—P2—C51	168.68 (13)
P2—C41—C42—C43	−176.90 (14)	C46—C41—P2—C8	96.84 (15)
C41—C42—C43—C44	0.5 (3)	C42—C41—P2—C8	−86.57 (14)
C42—C43—C44—C45	−0.6 (3)		
